# Chinese students’ filial piety beliefs and procrastination in mathematics learning: The mediating role of academic emotions

**DOI:** 10.3389/fpsyg.2023.1050259

**Published:** 2023-03-06

**Authors:** Meng Guo, Yiming Cao, Xiang Hu

**Affiliations:** ^1^School of Mathematical Sciences, Beijing Normal University, Beijing, China; ^2^School of Education, Renmin University of China, Beijing, China

**Keywords:** filial piety beliefs, enjoyment, anxiety, procrastination, mathematics learning

## Abstract

This study examined the relations between Chinese students’ filial piety beliefs and mathematics procrastination and the mediating role of academic emotions in the relations. Analysis of data on 1,476 primary school students in China with structural equation modeling revealed that students’ reciprocal and authoritarian filial piety beliefs were positively related to academic enjoyment and anxiety, respectively. Students’ procrastination in mathematics learning was positively related to anxiety and authoritarian filial piety beliefs and had negative associations with enjoyment and reciprocal filial piety beliefs. The bootstrap analysis results confirmed the mediating role of anxiety in the relation between authoritarian filial piety beliefs and procrastination. Reciprocal filial piety beliefs had negative indirect relationship with procrastination *via* enjoyment. The results were explained from a socio-cultural perspective. The theoretical contributions and practical implications are discussed.

## Introduction

Academic procrastination is a problematic behavior in student learning associated with a series of maladaptive learning outcomes such as poor academic performance (Moon and Illingworth, 2005; Kim and Seo, 2015) and less use of cognitive learning strategies (Wolters, 2003). Although academic procrastination has been extensively studied, extant research has overwhelmingly focused on Western populations (Klassen et al., 2008; Burnam et al., 2014). Preliminary studies found cross-cultural differences of students’ absolute level of academic procrastination and its consequences (e.g., Klassen et al., 2010), calling for more research of academic procrastination among non-Western samples such as East Asians (Jin et al., 2019; Yang et al., 2019).

Given the maladaptive role of procrastination in student learning, much attention has been paid to examine what contributes to students’ academic procrastination. Parent–child interaction has been found to be a critical factor affecting students’ procrastination behaviors (Ma et al., 2011; Zakeri et al., 2013; Chen et al., 2022). Filial piety beliefs (guiding children to repay, honor, and care for their parents, and unconditionally obey parents’ requirements; Ho, 1994) reflect Chinese students’ perceptions and beliefs of parent–child relationship and interaction (Yeh and Bedford, 2004; Chen, 2014). In Chinese societies, filial piety means that children should work hard to pursue for academic success to repay their parents and honor families (Tao and Hong, 2014), which may decrease students’ procrastination in learning process. However, few empirical studies have been conducted to investigate the relation between students’ filial piety beliefs and procrastination behaviors. This is important to understand cultural and family antecedents of students’ academic procrastination in Chinese societies.

Filial piety beliefs have been found to influence students’ academic outcomes, such as view of learning (Chen and Wong, 2014) and achievement (Chen, 2014). Academic emotions may also be shaped by students’ filial piety beliefs. According to control-value theory (Pekrun, 2019), students’ academic emotions are influenced by appraised and broader environmental antecedents. Filial piety beliefs reflect parent–child interaction from children’s perspectives, which may serve as a family antecedent of academic emotions. Children who tend to obey to parents’ authority (e.g., authoritarian filial piety) may have higher level of academic anxiety. As mathematics is a critical school subject for students’ future academic and career success in China, parents place high emphasis on their children’s mathematics learning and have high expectation on children’s mathematics performance (Cao et al., 2007). Thus, filial Chinese students may tend to internalize parents’ beliefs and endorse the value and importance of mathematics, and try to fulfill parents’ expectation, which may affect their emotions in mathematics learning. In other words, students’ filial piety beliefs may shape their appraisal for and emotions in mathematics.

Given the potential relationship between filial piety and emotions (Yeh, 2006) and between emotions and procrastination (Rahimi and Vallerand, 2021), academic emotions may serve as a mediator in the relations between filial piety beliefs and procrastination. In the Chinese context, filial students tend to value schools and engage more positive emotions in learning, which may create a protective mechanism to avoid procrastination. On the other hand, children who focus on family honor and obedience to parents are likely to experience more anxiety (Yeh, 2006), and thus engage in academic procrastination (Pychyl et al., 2000). In other words, students’ filial piety beliefs might influence their academic emotions and further shape their procrastination behaviors. However, there is a lack of empirical evidence testing this relation.

To fill in the aforementioned research gaps, this study aims to empirically examine the relations between Chinese students’ filial piety beliefs and their procrastination in mathematics learning and the mediational role of mathematics emotions in the relations.

## Literature review

### Filial piety beliefs

Filial piety is an important concept in Confucian culture, describing how children should interact with their parents (Ho and Lee, 1974; Yeh and Bedford, 2003). Filial piety requires children to love their parents by supporting and caring for parents, maintaining family honor, obeying parents’ decisions, and achieving parents’ wishes (Ho, 1994). Some research showed that filial piety was associated with maladaptive outcomes, including cognitive conservatism (Ho, 1996), neuroticism (Zhang and Bond, 1998), and less creativity (Ho, 1994). In contrast, other studies identified a positive role of filial piety, as it facilitated harmonious intergenerational relationships (Sung, 1995) and family cohesion (Cheung et al., 1994).

Given the contradictory findings on the role of filial piety, Yeh (2003) proposed the dual filial piety model (DFPM), which further divides filial piety into two dimensions: reciprocal and authoritarian filial piety. DFPM posits that reciprocal filial piety is associated with adaptive outcomes, while authoritarian filial piety is associated with maladaptive outcomes (Yeh, 2003). Reciprocal filial piety comprises “emotionally and spiritually attending to one’s parents out of gratitude for their efforts in having raising one, and physical and financial care for one’s parents as they age and when they die for the same reason,” while authoritarian filial piety encompasses “suppressing one’s own wishes and complying with one’s parents’ wishes because of their seniority in physical, financial or social terms, as well as continuing the family lineage and maintaining one’s parents’ reputation because of the force of role requirements” (Yeh and Bedford, 2003, p. 216). Existing literature has largely support DFPM in that reciprocal filial piety is associated with desirable outcomes such as openness (Yeh and Bedford, 2003) and better interpersonal relationships (Yeh and Bedford, 2004), whereas authoritarian filial piety is related to maladaptive outcomes, like performance-avoidance goals (Chen, 2016), neuroticism (Yeh and Bedford, 2003), and anxiety (Yeh, 2006). Therefore, this study followed DFPM (Yeh, 2003) to conceptualize filial piety as comprising both reciprocal and authoritarian filial piety.

### Filial piety and academic procrastination

Procrastination refers to the unnecessary delay in taking action, despite unavoidably undesirable results (Steel, 2007). Academic procrastination is seen as an undesirable learning tendency, as it was always related to such maladaptive learning outcomes as high levels of anxiety and stress (Chu and Choi, 2005; Spada et al., 2006), less use of cognitive learning strategies (Wolters, 2003), and poor academic performance (Moon and Illingworth, 2005; Kim and Seo, 2015). Given the maladaptive role of procrastination in student learning, it is essential to identify potential antecedents of procrastination (Steel and Klingsieck, 2016; Rahimi and Vallerand, 2021). Previous studies have found that procrastination was influenced by individual personality traits (e.g., conscientiousness; Steel and Klingsieck, 2016) and motivation (e.g., goal orientations; Howell and Watson, 2007), teacher-related factors (e.g., teachers’ clear expectations; Schraw et al., 2007), and so forth.

Researchers have found that East Asian culture shapes students’ learning process (Leung, 2006; Hu et al., 2018; Guo et al., 2022). However, informative as the extant literature is, few studies have empirically examined whether and how cultural background might influence academic procrastination. Further, it is noteworthy that research on academic procrastination has mostly surveyed college students (Rabin et al., 2011; Kim and Seo, 2015), while younger students’ academic procrastination has been under-investigated (Xue et al., 2021). To fill in the research gaps, this study focused on one potential cultural antecedent of academic procrastination—students’ filial piety beliefs—and examined the relation between filial piety beliefs and procrastination in mathematics learning among primary school students in China.

Exiting evidence on parental influence on children’s academic procrastination provides support for our hypothesis on the relation between filial piety and academic procrastination. Previous studies have found that authoritarian parenting style positively predicted children’s academic procrastination (Zakeri et al., 2013; Soysa and Weiss, 2014; Chen et al., 2022). For instance, by investigating 743 Chinese college students, Chen et al. (2022) identified that students who perceived authoritarian parenting style were more inclined to focus on mistakes and thus procrastinated learning tasks. Given authoritarian filial piety beliefs reflect students’ perception of authoritarian parent–child interaction (Chen, 2014), it is also likely to increase children’s academic procrastination. Specifically, students with authoritarian filial piety tend to restrain their own wishes and obey their parents’ academic requirements (Yeh, 2006), which may hurt their individual autonomy (Sun et al., 2019) in the learning process, and further lead to more academic procrastination. Therefore, students’ authoritarian filial piety is hypothesized to increase their procrastination in mathematics learning.

Regarding reciprocal filial piety, there is also a lack of research on its association with academic procrastination in mathematics learning. However, existing literature have confirmed its beneficial role in student personal development and academic learning (Yeh, 2006). A study of 750 junior high school students revealed that students’ reciprocal filial belief positively predicted their autonomy need satisfaction and academic performance in mathematics and reading (Zhou et al., 2020). Indeed, studying hard to achieve academic success is regarded as an important way to repay parents in China (Leung, 2006; Tao and Hong, 2014). Students who have the desire to repay their parents (i.e., reciprocal filial piety) tend to be hardworking at school and less academically procrastinated. Therefore, we hypothesize that reciprocal filial piety will negatively predict academic procrastination.

### The mediating role of academic emotions

The importance of academic emotions students experienced in school settings has long been recognized by educators. Academic emotions refer to “emotions tied directly to achievement activities or achievement outcomes” (Pekrun, 2006, p. 317). Pekrun’s (2006) control-value theory (CVT) posits that students’ academic emotions are influenced by appraised and broader environmental antecedents, and influence learning outcomes such as achievement, motivation to learn, and self-regulation. Filial piety, as an essential cultural aspect in China, might influence students’ academic emotions, which in turn affects procrastination in mathematics learning. That is, academic emotions may play a mediating role on the relation between filial piety and academic procrastination. However, there are, to the best of our knowledge, few studies have empirically tested the mediating effect of academic emotions on the relation, which is a focus of our study. Notably, we specifically focus on two academic emotions (i.e., enjoyment and anxiety) as they have been widely studied and demonstrated importance in Chinese students’ learning process (Jiang and Dewaele, 2019; Guo et al., 2020).

The relation between academic emotions and academic procrastination has been well-documented in literature (Spada et al., 2006; Rahimi and Vallerand, 2021). Positive academic emotions (e.g., enjoyment) can serve as a protective factor for academic procrastination (Rahimi and Vallerand, 2021), while negative emotions were found to trigger more academic procrastination (Pychyl et al., 2000; Rahimi and Vallerand, 2021). Rahimi (2019) conducted a three-phase longitudinal study and found a reciprocal positive relationship of enjoyment and anxiety with academic procrastination. Therefore, we hypothesize that mathematics enjoyment and anxiety will have a positive and negative association with mathematics procrastination, respectively.

The relation of filial piety with academic enjoyment and anxiety has been under-investigated. Preliminary evidence, however, support the role of filial piety in students’ emotional process (Yeh, 2006). By investigating senior and junior high school students in Chinese Taipei, Yeh (2006) found that students with reciprocal filial piety perceived less level of depression and anxiety, while students’ authoritarian filial piety triggered their experience of anxiety. Thus, we hypothesize that reciprocal and authoritarian filial piety beliefs negatively and positively predict mathematics anxiety, respectively. Further, extant evidence on the relation between filial piety and academic motivation might shed light on the relation between filial piety and academic enjoyment. For instance, Chen (2016) found that reciprocal filial piety positively predicted Chinese students’ mastery goals. Students with mastery goals always experienced more positive emotions (e.g., enjoyment; Lee et al., 2003; Pekrun et al., 2009). Thus, this study hypothesizes that students’ reciprocal filial piety will positively predict enjoyment. Authoritarian filial piety beliefs require children to unconditionally follow parents’ arrangements and obey parental authority, which may hinder students’ individual autonomy in the learning process (Sun et al., 2019). According to self-determination theory, autonomy serves as an important factor facilitating the development of intrinsic motivation (Niemiec and Ryan, 2009). Researchers have identified the positive relationship between intrinsic motivation and enjoyment (Gråstén et al., 2012). Therefore, authoritarian filial piety is hypothesized to negatively predict mathematics enjoyment.

To sum up, extant studies have identified associations between filial piety and academic emotions (Yeh, 2006) and between academic emotions and procrastination (Rahimi and Vallerand, 2021), indicating a potential mediating role of academic emotions in the relation between filial piety and procrastination.

### The present study

Drawing on the control-value theory of academic emotions, we advanced an indigenous psychological perspective to examine the relations between Chinese students’ filial piety—an essential cultural belief for Chinese people—with their academic emotions (i.e., math enjoyment and anxiety) and procrastination in mathematics learning. Based on previous studies on filial piety, emotions, and academic procrastination (Yeh, 2006; Chen, 2016; Rahimi and Vallerand, 2021), this study hypothesizes that students’ filial piety has a significant relationship with academic emotions and further links to procrastination in mathematics learning. The specific hypothesis in the study is presented below.

*Hypothesis 1*: Authoritarian filial piety beliefs will be related to a higher level of mathematics anxiety and less enjoyment.

*Hypothesis 2*: Reciprocal filial piety beliefs are expected to be positively and negatively associated with mathematics enjoyment and anxiety, respectively.

*Hypothesis 3*: Mathematics anxiety and enjoyment will have a positive and negative association with academic procrastination, respectively.

*Hypothesis 4*: Reciprocal and authoritarian filial piety beliefs will be negatively and positively related to academic procrastination, respectively.

## Methods

### Participants and procedure

This study involved 1,476 Fourth to Sixth Grade primary school students (Mage = 10.82 years, SD = 0.96) in a city of Guizhou Province, China. Of these students, 48.0% were boys, 51.5% were girls, and 0.5% were missing gender information. After consulting with local experienced teachers, we invite each school with high, medium, and low teaching quality, respectively, in the city to participated into the study, resulting in totally 3 participating schools. With the help of teachers in the schools, the first author administered the questionnaire to students in class. Before data collection, ethics approval was acquired. Students who agreed to take part in this study filled in a consent form and then completed a self-reported questionnaire on filial piety and academic emotions.

### Measures

#### Filial piety

The filial piety scale (Yeh, 2003) was used to measure both reciprocal filial piety (4 items; α =0.813; e.g., “Children should support their parents to make them live better”) and authoritarian filial piety (4 items; α =0.769; e.g., “Children should do what their parents ask”), on a 5-point Likert scale.

#### Academic emotions

Measures of mathematics enjoyment and anxiety were adapted from the enjoyment and anxiety subscale in the Achievement Emotions Questionnaire (Pekrun et al., 2011) and further revised to focus on mathematics learning. Four and three items were employed to measure mathematics enjoyment (α = 0.846; 4 items; e.g., “Mathematics class makes me feel happy”) and anxiety (α = 0.795; 3 items; e.g., “I am worried that mathematics class will be very difficult”), respectively.

#### Procrastination

Four items were used to measure student procrastination in mathematics learning (α = 0.854; 4 items, e.g., “I often put off finishing my math homework”), which were adapted from the procrastination scale in the study of Wolters (2003).

#### Background variables

Student gender (boy = 0; girl = 1), grade (Grade 4 = 1; Grade 5 = 2; Grade 6 = 3) and parent highest educational level (primary school = 1; secondary School = 2; high school = 3; university = 4; master’s degree or above = 5) were considered as control variables in the analysis.

### Data analysis

Before formal analyses, we imputed missing values of included variables using expectation maximization (EM) algorithm in SPSS software (Version 25.0). Structural equation modeling (SEM) was used to model the relation between students’ filial piety beliefs, academic emotions, and procrastination. The comparative fit index (CFI) > 0.90 (Bentler, 1990), the Tucker-Lewis index (TLI) > 0.90, and the root mean squared error of approximation (RMSEA) < 0.08 (Browne and Cudeck, 1992) indicated acceptable model fit. Bootstrap methods with 5,000 resamples were conducted to test the mediation effects of academic emotions in the relation between filial piety beliefs and procrastination (Hayes, 2013) using Mplus software (Version 8.0).

## Results

### Preliminary analysis

Results of descriptive statistics and correlations among variables are shown in Table 1. Authoritarian filial piety was positively correlated with reciprocal filial piety (*r* = 0.224, *p* < 0.01). Mathematics enjoyment had positive correlations with reciprocal (*r* = 0.358, *p* < 0.01) and authoritarian filial piety (*r* = 0.109, *p* < 0.01). Mathematics anxiety was positively related to authoritarian filial piety (*r* = 0.064, *p* < 0.05). Procrastination had positive correlations with authoritarian filial piety (*r* = 0.067, *p* < 0.01) and anxiety (*r* = 0.236, *p* < 0.01) and had negative correlations with reciprocal filial piety (*r* = −0.227, *p* < 0.01) and enjoyment (*r* = −0.260, *p* < 0.01).

**Table 1:** Descriptive statistics, correlations, and internal consistency of the main variables in this study.

	RFP	AFP	En	An	Pr	PE	Gr	SG
RFP	1							
AFP	0.224^**^	1						
En	0.358^**^	0.109^**^	1					
An	0.009	0.064^*^	−0.126^**^	1				
Pr	−0.227^**^	0.067^**^	−0.260^**^	0.236^**^	1			
PE	0.040	−0.162^**^	0.094^**^	−0.094^**^	−0.080^**^	1		
Gr	−0.032	−0.224^**^	−0.091^**^	0.140^**^	0.009	−0.066^*^	1	
SG	0.034	−0.021	−0.040	0.103^**^	−0.108^**^	−0.075^**^	0.038	1
M	4.595	3.547	3.967	3.345	1.646	2.540	1.957	0.517
SD	0.525	0.876	0.780	1.079	0.769	0.956	0.802	0.500
α	0.813	0.769	0.846	0.795	0.854	/	/	/

RFP, Reciprocal filial piety; AFP, Authoritarian filial piety; Enjoyment, En; Anxiety, An; Procrastination, Pr; PE, parental highest educational level; Gr, Grade; SG, Student gender. ^*^p < 0.05, ^**^p < 0.01.

### Structural equation modeling

The SEM results showed that the hypothesized model fit the data well [*X*^2^(*df*) = 529.101 (184), *X*^2^/*df* = 2.876, *RMSEA* = 0.036 with 90% *CI* [0.032, 0.039], *CFI* = 0.969, *TLI* = 0.962]. The factor loading of each item was significant, ranging from 0.493 to 0.818. The variance explained by the model was 0.165 for enjoyment, 0.056 for anxiety, and 0.211 for academic procrastination, respectively.

**Figure 1:**
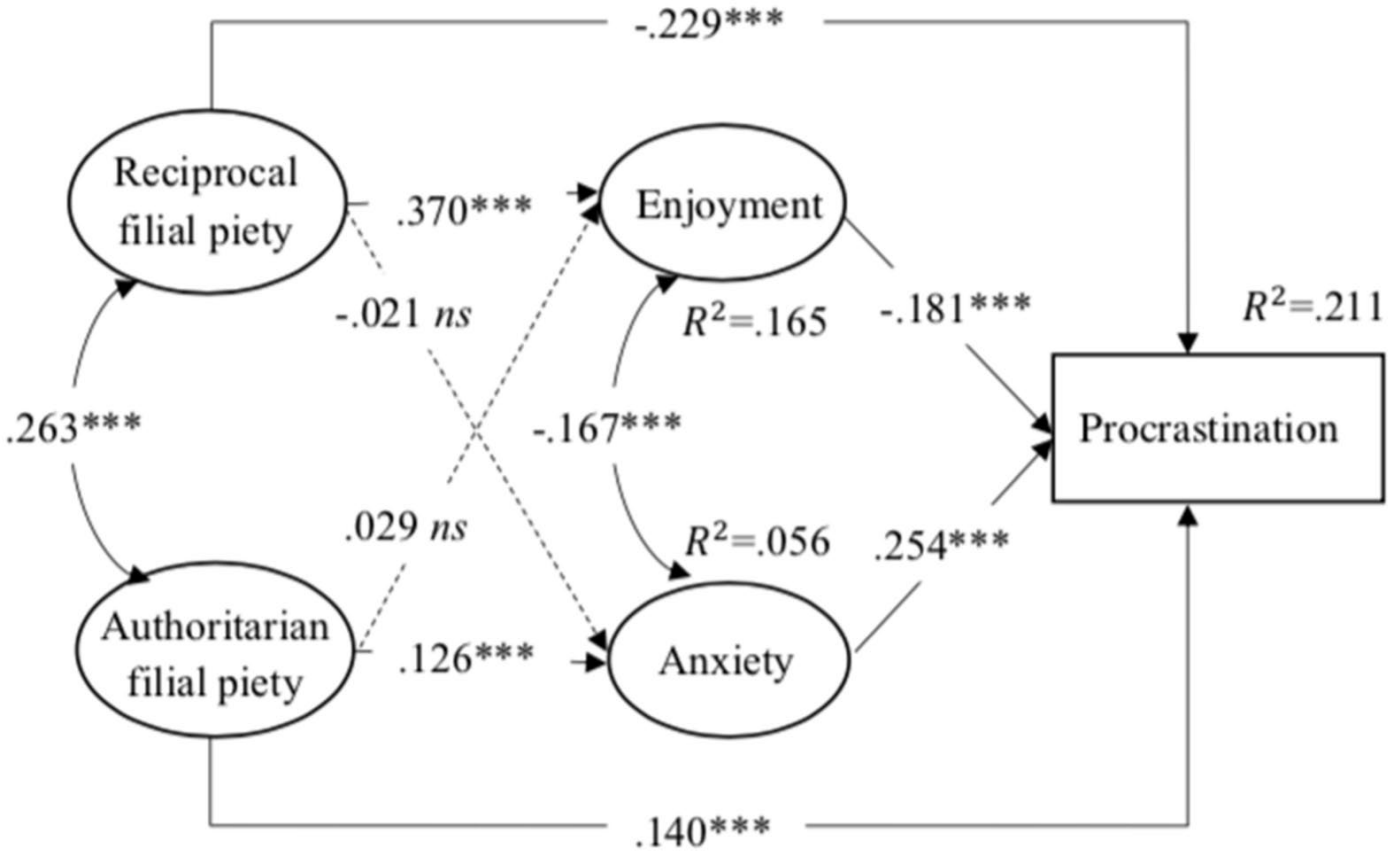
The relationship between Chinese students’ filial piety beliefs, academic emotions and academic procrastination. Non-significant paths and covariables were not included in the figure for clarity. ^***^p < 0.001.

As shown in Figure 1, reciprocal filial piety was positively associated with enjoyment ( β=0 .370, *p* < 0.001). Authoritarian filial piety was positively related to anxiety ( β=0 .126, *p* < 0.001). Academic procrastination was negatively predicted by both enjoyment ( β= −0.181, *p* < 0.001) and reciprocal filial piety ( β= -0.229, *p* < 0.001). Authoritarian filial piety ( β=0 .140, *p* < 0.001) and anxiety ( β=0 .254, *p* < 0.001) had a positive association with academic procrastination.

### Mediation tests

We checked the potential mediating roles of academic emotions in the relation between filial piety and procrastination *via* bootstrap methods (see Table 2). The results indicated that reciprocal filial piety had a direct negative relationship with student academic procrastination ( β= −0.229, *p* < 0.001; 95%CI [−0.300, −0.159]) and indirect effects *via* enjoyment ( β= −0.067, *p* < 0.001; 95%CI [−0.098, −0.040]). The authoritarian filial piety was positively related to procrastination *via* anxiety ( β=0 .032, *p* = 0.002; 95% CI [0.014, 0.054]). The direct positive effect of authoritarian filial piety on procrastination was also significant ( β=0 .140, *p* < 0.001; 95%CI [0.076, 0.203]).

**Table 2:** Standardized total effects, direct effect, and indirect effect on procrastination.

	Point estimate			Bootstrapping BC 95%CI	
	Standardized *β*	S.E.	*p*	Lower	Upper
**Effects from RFP to Pr**					
Direct effects	−0.229^***^	0.036	0.000	−0.300	−0.159
Indirect effects					
RFP-En-Pr	−0.067^***^	0.014	0.000	−0.098	−0.040
RFP-An-Pr	−0.005	0.008	0.494	−0.020	0.010
Total effects	−0.301^***^	0.034	0.000	−0.369	−0.234
**Effects from AFP to Pr**					
Direct effects	0.140^***^	0.032	0.000	0.076	0.203
Indirect effects					
AFP-En-Pr	−0.005	0.007	0.453	−0.021	0.007
AFP-An-Pr	0.032^**^	0.010	0.002	0.014	0.054
Total effects	0.167^***^	0.033	0.000	0.101	0.230

RFP, Reciprocal filial piety; AFP, Authoritarian filial piety; Enjoyment, En; Anxiety, An; Procrastination, Pr. ^**^p < 0.01, ^***^p < 0.001.

## Discussion

The aim of this study was to examine the relations between students’ filial piety and academic procrastination in mathematics learning and the mediating role of mathematics enjoyment and anxiety in the relations. Results were all consistent with our hypotheses. We found that reciprocal filial piety had a direct positive relationship with procrastination. A plausible explanation is that students with reciprocal filial piety are more inclined to focus on mastering knowledge and improving competence (Chen, 2016), which can further minimize procrastination behaviors (Wolters, 2004). This is consistent with King and McInerney’s (2019) findings that students who had the desire to repay and support their parents tended to have less disengagement. Another possibility is that students who are grateful to their parents have a higher level of self-esteem (Yan and Chen, 2018), which can prevent procrastination behaviors (Hajloo, 2014).

As hypothesized, authoritarian filial piety was positively related to procrastination in mathematics learning. Researchers have found that students who endorse authoritarian filial piety tend to hold an entity view of learning (i.e., intelligence is fixed and unchangeable; Chen and Wong, 2014), which may in turn lead to more procrastination (Rickert et al., 2014). Another plausible explanation is that students who focus on gaining family honor and meeting parents’ requirements may worry about possible academic failure and adopt performance-avoidance goals (Chen, 2016), and thus have more academic procrastination (Wolters, 2004). Also, authoritarian filial piety may be detrimental to the development of students’ individual autonomy (Sun et al., 2019), which may hurt their intrinsic motivation (Ryan and Deci, 2000) and thus lead to procrastination in the learning process (Senécal et al., 1995).

We found that reciprocal filial piety beliefs were positively related to students’ enjoyment of mathematics learning, indicating that students who had the desire to repay and love parents experienced more academic enjoyment. Students with reciprocal filial piety tend to have higher levels of self-esteem (Yan and Chen, 2018), which may further increase their academic enjoyment (Cheng and Furnham, 2003). Previous studies have found that students’ reciprocal filial piety can promote their endorsement of mastery goals (Chen, 2016), which further trigger their enjoyment of learning (Pekrun et al., 2009). Another possible explanation is that students who endorse reciprocal filial piety tend to have a close and intimate relationship with their parents. These students may perceive more relatedness with their parents. Based on self-determination theory, relatedness can foster students’ intrinsic motivation (Niemiec and Ryan, 2009) and further increase students’ enjoyment experience in the learning process (Gråstén et al., 2012).

Authoritarian filial piety beliefs were found to have a positive relationship with students’ academic anxiety, which is consistent with previous studies (Yeh, 2006; Rahimi and Vallerand, 2021). It suggests that students who emphasize family reputation and obey parental authority tend to experience more anxiety. These students may worry about the possible negative effects of academic failure on their families (e.g., losing their parents’ faces, and failing to meet parental expectations), which may lead to a higher level of anxiety in learning. Previous studies have confirmed that authoritarian filial piety increased students’ adoptions of performance-avoidance goals (Chen, 2016), which can trigger students’ experience of anxiety (Pekrun et al., 2009).

Our findings revealed that students’ mathematics enjoyment was negatively related to mathematics procrastination. If students experienced enjoyment in the learning process, they may tend to continue pursuing the positive experience in the learning tasks, which further facilitates academic engagement and avoid academic procrastination. The negative relationships between positive emotions and procrastination were also found by some previous researchers (Balkis and Duru, 2016; Rahimi, 2019). For instance, Rahimi and Vallerand (2021) identified that students with positive emotions in their studies tended to have less procrastination in their academic assignments.

As hypothesized, students who experienced a higher level of mathematics anxiety were more inclined to procrastinate in the learning process. The close relationship between negative emotions and academic procrastination was also confirmed in many previous studies (Pychyl et al., 2000; Spada et al., 2006; Balkis and Duru, 2016; Rahimi, 2019; Rahimi and Vallerand, 2021). When students perceived anxiety in the learning process, they may delay the completion of academic tasks to escape from the negative emotions evoked by the assignment.

This study confirmed the mediational role of academic emotions between filial piety beliefs and procrastination in mathematics learning. Students who have gratitude to parents and hope to repay parents tended to experience more enjoyment in the learning process and further had less academic procrastination, while students who focused on obedience to parents and maintaining family honor were more likely to experience anxiety and thus engaged more in academic procrastination.

Our findings have significant implications for educational practices. Parents are suggested to establish intimate relationships and affection with their children, which can develop children’s reciprocal filial piety (Yeh et al., 2013) and further promote the experience of positive emotions and reduce undesirable learning outcomes. Based on the negative nature of authoritarian filial piety in students’ mathematics learning, parents need to avoid authoritarian parent–child interactions and reduce parental control in family contexts. Parents are suggested to create a democratic and harmonious family climate, which can minimize children’s negative emotions and avoid them from engaging in academic procrastination.

Some limitations should be noted in the present study. First, this study used a cross-sectional design, so the results cannot reveal causality. Thus, caution should be made in explaining the findings in this study. Longitudinal research design is needed in future studies to check the causal relationship. Second, this study was only conducted with a sample of primary school students from three schools in China. The relationship between filial piety and academic emotions and procrastination may vary across different regions and educational levels. Further studies can check and expand our findings by investigating students in other countries and regions and other grade levels (e.g., secondary school and college). Third, this study only collected students’ self-reported questionnaire data. Qualitative research methods (e.g., interview and observation) are needed to deeply explore the influence of filial piety on students’ mathematics learning process and the potential reasons behind their relationship. Fourth, the control variables in this study only include student gender, grade, and parent educational level. In fact, students’ prior mathematics achievement may significantly affect their emotion and procrastination. However, the present study did not control the influence of students’ mathematics performance when examining the relationship between filial piety, emotions, and procrastination. Future studies need to take students’ mathematics performance as a control variable in the relationship.

## Conclusion

This study confirmed the association between students’ filial piety beliefs and mathematics procrastination and the mediating role of mathematics enjoyment and anxiety in the association. The results suggested that students with reciprocal filial piety tended to have fewer procrastination behaviors. The reciprocal filial piety triggered more academic enjoyment, which may establish a protective mechanism against procrastination. We also identified that students who endorsed authoritarian filial piety were inclined to have more procrastination in mathematics learning. Students with authoritarian filial piety experienced more anxiety, which further led to more academic procrastination. These results expand our understanding of the relation between filial piety beliefs, mathematics emotions, and mathematics procrastination, which helps to unpackage the role of filial piety and academic emotions in Chinese students’ mathematics learning process.

## Data availability statement

The raw data supporting the conclusions of this article will be made available by the authors, without undue reservation.

## Ethics statement

The studies involving human participants were reviewed and approved by Faculty of Education, The University of Hong Kong. Written informed consent to participate in this study was provided by the participants’ legal guardian/next of kin.

## Author contributions

MG, YC, and XH contributed to conception and design of the study. MG collected the data and performed the statistical analysis. MG and XH wrote the first draft of the manuscript. All authors contributed to the article and approved the submitted version.

## Funding

This research was funded by Beijing Normal University Postdoctoral Research Fund (no. 110321001) and China Postdoctoral Science Foundation (no. 2022M710418).

## Conflict of interest

The authors declare that the research was conducted in the absence of any commercial or financial relationships that could be construed as a potential conflict of interest.

## Publisher’s note

All claims expressed in this article are solely those of the authors and do not necessarily represent those of their affiliated organizations, or those of the publisher, the editors and the reviewers. Any product that may be evaluated in this article, or claim that may be made by its manufacturer, is not guaranteed or endorsed by the publisher.
